# Limited Accuracy of Mechanism, Glasgow Coma Scale, Age, And Pressure (MGAP) in Polytraumatic Patients: A Cross-sectional Study -

**DOI:** 10.31661/gmj.vi.3552

**Published:** 2025-06-25

**Authors:** Marzieh Meraji, Hamed Tabesh, Mahboobe Tashakkori, Mahdi Foroughian, Elham Pishbin, Morteza Talebi Doluee

**Affiliations:** ^1^ Department of Health Information Technology, School of Paramedical Sciences, Mashhad University of Medical Sciences, Mashhad, Iran; ^2^ Department of Medical Informatics, School of Medicine, Mashhad University of Medical Sciences, Mashhad, Iran; ^3^ Department of Emergency Medicine, Faculty of Medicine, Mashhad University of Medical Sciences, Mashhad, Iran

**Keywords:** Mortality, Trauma, Multiple Traumata, ISS, MGAP

## Abstract

**Background:**

Trauma, an external assault’s corporeal aftermath, manifests as wounds or injuries, breaching or not breaching the skin, wrought by environmental forces upon the human, and is considered One of the world’s foremost public health challenges intertwines complex societal, environmental, and medical issues. In order to evaluate the severity of trauma, it seems necessary to have a quantitative scale that can be measured. Therefore, the aim of this study is to look into the predictive power of two scoring indices, The Injury Severity Score (ISS) and MGAP, in patients with multiple traumas.

**Materials and Methods:**

This research is a cross-sectional type and was conducted on trauma patients, aged 18 and over, with at least two traumas, hospitalized for over 24 hours in Taleghani Hospital in Mashhad in the period of one year from October 2020 to October 2021. For ISS, the Abbreviated Injury Scale (AIS) scores for affected organs in six body areas were determined, and the three highest AIS scores were calculated. For the MGAP score, data on injury mechanism, age, Glasgow Coma Scale (GCS), and systolic blood pressure were extracted. A chi-square test was conducted to ascertain the correlations between categorical variables and the status of survival. Additionally, Positive Triage Accuracy was measured and compared for ISS and MGAP.

**Results:**

In this research, 699 cases were investigated. Among the patients, 567 (81%) were male and 132 (19%) were female. Also (92%) 641 patients were discharged alive and (8%) 58 patients died. Among the 114 patients admitted to the ICU, 45 (39%) died. Cases mislabeled as medium or low risk that experienced death happened in 7 cases (of 598 available records; 1.17%) based on the ISS risk stratification system with 98.18% Positive Triage Accuracy and 44 cases (of 698 available records; 6.30%), based on the MGAP risk stratification system with 32.75% positive triage accuracy.

**Conclusion:**

The findings show the robust predictive power of ISS, with a low mislabeling rate and high triage accuracy. Conversely, the MGAP system demonstrated a higher mislabeling rate, suggesting that its application may yield incorrect results in certain situations and settings. This shows the need for cautious consideration when applying the MGAP risk stratification system, as its performance may not be universally applicable across all scenarios.

## Introduction

Trauma is among the most common causes of death worldwide and the second cause of death of young people in Iran, as well as one of the four causes of death in developing countries [1]. Every year, 5.7 million deaths occur due to various traumatic injuries. It is estimated that in 2020, about 20% of the worldwide health load was related to traumatic injuries [2].

Traffic accidents are the cause of 26% of deaths in the world [3]. According to the report of the World Health Organization (WHO), 1.24 million people (18 per 100,000 people) die due to traffic accidents every year [4]. To appropriately manage trauma patients, a quantitative scale would be necessary in order to evaluate the severity of trauma. Since several years ago, various scales have been used to determine the severity of trauma in trauma patients [5].

The Shock Index was developed in 1967 and the Revised Trauma Score (RTS) in 1981 by Champion. The revised trauma score has been most commonly used to triage and predict the mortality of patients in emergency units [6]. In 1971, the Abbreviated Injury Scale (AIS) system was developed by the American Society of Surgeons and the Committee on Medical Aspects of Driving Accidents to evaluate the severity of blunt trauma injuries (5). In this system, based on the severity of the damage to the organ, a score of 1 to 6 is given. The Injury Severity Score (ISS) is determined by squaring the AIS scores of the three most critically injured anatomical regions. The Exponential Injury Severity Score (EISS) was created in 2014 by modifying the AIS system. EISS has been reported as a better predictor of survival in multiple trauma patients [7].

Also, another adjusted score called Military Injury Severity (MISS) is used to predict war-related deaths [8]. Recently, two modified scoring systems including variables of MMGAP and the Glasgow Coma Scale, Age, And Pressure (GAP) has been developed to predict survival in trauma patients (1). MGAP provides a well-rounded evaluation of traumatic injury [9]. The MGAP score’s versatility makes it applicable across diverse trauma cases [10]. Studies have indicated that the MGAP, along with the GAP (GCS, Age, and Pressure) score, can be superior to other scoring systems, such as the Revised Trauma Score (RTS), in predicting mortality among trauma patients [11].

But, when being compared with other scoring systems, it showed less accuracy than the Trauma Related Injury Severity Score (TRISS) for mortality prediction [11]. Exposure to multiple traumatic events has been consistently associated with increased severity of trauma-related outcomes. Understanding how ISS and MGAP perform in predicting mortality provides valuable information about the clinical utility of these scoring systems. By evaluating the predictive capabilities of ISS and MGAP, the study aims to contribute to the enhancement of patient outcomes. The outcomes of this study can serve as a guide for clinicians, trauma teams, and healthcare institutions when making decisions about the adoption and utilization of trauma scoring systems.

## Materials and Methods

This cross-sectional research was conducted at Taleghani Hospital in Mashhad over the course of one year, spanning from October 2020 to October 2021. The city of Mashhad houses three specialized trauma centers—Shahid Hashminejad Hospital, Shahid Ghayab Hospital, and Shahid Taleghani Hospital—providing the backdrop for this study. The research unfolded in three distinct phases: data collection, indicator computation, and the subsequent assessment and validation of scoring indicators.

Shahid Taleghani Hospital in Mashhad was selected as the research environment. Data were meticulously extracted from patient files and the Hospital Information System (HIS) using a predetermined data extraction form. The research population comprised files of multiple trauma patients, adhering to specific inclusion and exclusion criteria. Inclusion criteria encompassed inpatients, trauma patients hospitalized for over 24 hours, individuals aged 18 and older, and multiple trauma patients with at least two traumas (both penetrating and closed).

Exclusion criteria included the absence of a triage sheet in the file, pregnant individuals, and those with psychiatric disorders, poisonings, various types of cancers, burns, and underlying diseases.

### Sample Size Determination

The minimum sample size, estimated at approximately 10% of the research population, was determined to be around 700 individuals. One case, involving elective admission and a lack of patient triage upon arrival, was excluded, resulting in a final study sample of 699 cases.

### Scoring Systems

The Injury Severity Score (ISS) computation involved determining the Abbreviated Injury Scale (AIS) score for affected organs in six body areas. The three injuries with the highest AIS scores were then squared, summed, and categorized into five severity levels—mild, moderate, severe, very severe, and critical.

The MGAP score calculation extracted information on injury mechanism, age, Glasgow Coma Scale (GCS), and systolic blood pressure from patient files. The scoring involves assigning points based on specific criteria: for the mechanism of injury, blunt mechanisms such as falls or motor vehicle collisions receive 0 points, while penetrating mechanisms like gunshot or stab wounds receive 7 points. Glasgow Coma Scale scores are categorized, with a GCS of 15 earning 0 points, GCS 13-14 earning 7 points, GCS 12 earning 15 points, GCS 9-11 earning 20 points, GCS 6-8 earning 25 points, and GCS 3-5 earning 30 points. Age categories contribute points, with age under 60 receiving 0 points, age 60-69 earning 10 points, age 70-79 earning 20 points, and age 80 or older earning 30 points. Systolic blood pressure (SBP) also factors in, with SBP>89 mmHg receiving 0 points, SBP 76-89 mmHg earning 5 points, SBP 50-75 mmHg earning 10 points, and SBP<50 mmHg earning 20 points. The total MGAP score is the sum of these points, ranging from 0 to 100. Risk groups are then determined: Low-Risk (MGAP score 23-29), Medium-Risk (MGAP score 15-22), and High-Risk (MGAP score<15).

### Data Analysis

All data were anonymized and handled in accordance with ethical guidelines. Descriptive statistics were harnessed to distill the demographic and clinical profiles of the study populace. Frequencies and percentages were ascertained for categorical variables, while means and standard deviations were invoked for continuous variables.

The chi-square test was deployed to elucidate the relationships between categorical variables and survival status. Positive Triage Accuracy was used to measure the accuracy of the triage system in correctly identifying and prioritizing patients who require immediate attention or intensive care. It was calculated as the number of patients correctly triaged as intense or very intense divided by the total number of patients in those categories.

Positive Triage Accuracy=Correctly triaged as intense or very intense/Total number of patients in intense or very intense categories

## Results

**Table:** Table1. Demographic and Cinical Characteristics of Study Participants Stratified by Survival Status

		**survived**	**dead**	**total**	**P**
		n (%)	n (%)	n (%)	
	18-44	442(68.85%)	22(37.93%)	464(66.38%)	
**age**	45-60	127(19.78%)	20(34.48%)	147(21.03%)	<0.0001
	60 <	72(11.21%)	16(27.59%)	88(12.59%)	
**gender**	male	522(81.31%)	45(77.59%)	567(81.12%)	0.587
	female	119(18.54%)	13(22.41%)	132(18.88%)	
	3 to 5	19(2.96%)	28(48.28%)	47(6.72%)	<0.0001
**GCS**	6 to 12	18(2.8%)	5(8.62%)	23(3.29%)	
	13 to 15	603(93.93%)	25(43.1%)	628(89.84%)	
**race**	Iranian	605(94.24%)	57(98.28%)	662(94.71%)	0.336
	Foreign nationals	36(5.61%)	1(1.72%)	37(5.29%)	
	accident	405(63.08%)	43(74.14%)	448(64.09%)	0.237
**trauma reason**	Fall	146(22.74%)	10(17.24%)	156(22.32%)	
	other	90(14.02%)	5(8.62%)	95(13.59%)	
**injury mechanism**	penetrating	128(19.94%)	19(32.76%)	147(21.03%)	0.033
	Blunt	513(79.91%)	39(67.24%)	552(78.97%)	
	Level 1	26(4.05%)	26(44.83%)	52(7.44%)	<0.0001
**triage level**	Level 2	551(85.83%)	32(55.17%)	583(83.4%)	
	Level 3	62(9.66%)	0(0%)	62(8.87%)	
**ICU hospitalization**	Yes	69(10.75%)	45(77.59%)	114(16.31%)	<0.0001
	no	572(89.1%)	13(22.41%)	585(83.69%)	
	Partial recovery	595(92.68%)	0(0%)	595(85.12%)	<0.0001
	Full recovery	1(0.16%)	0(0%)	1(0.14%)	
**outcome**	Follow up	6(0.93%)	0(0%)	6(0.86%)	
	dead	0(0%)	53(91.38%)	53(7.58%)	
	Brain death	0(0%)	5(8.62%)	5(0.72%)	
	Low risk	23(3.58%)	0(0%)	23(3.29%)	<0.0001
	medium	190(29.6%)	7(12.07%)	197(28.18%)	
**ISS**	intense	198(30.84%)	17(29.31%)	215(30.76%)	
	very intense	78(12.15%)	20(34.48%)	98(14.02%)	
	critical	54(8.41%)	11(18.97%)	65(9.3%)	
	high risk	5(0.78%)	14(24.14%)	19(2.72%)	<0.0001
**MGAP scale**	medium	60(9.35%)	25(43.1%)	85(12.16%)	
	low risk	575(89.56%)	19(32.76%)	594(84.98%)	

In this research, 699 cases were investigated. Among the patients, 567 (81%) were male and 132 (19%) were female. Also (92%) 641 patients were discharged alive and (8%) 58 patients died. The chi-square test indicates that the distribution of survival status across age categories is statistically significant (P<0.0001). Specifically, among patients aged 18-44, 95% survived. In the 45-60 age group, 86% survived, and for those aged 60 and above, 82% survived. Gender, trauma reason, and race did not show significant association with mortality (P>0.05).

**Figure-1:**
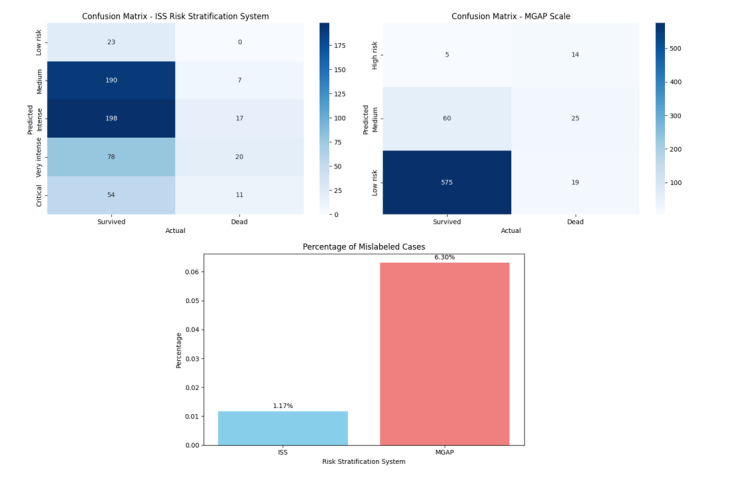
Figure 1. Risk stratification performance for mortality prediction: (Top-left) Confusion matrix for Injury Severity Score (ISS); (Top-right) Confusion matrix for Modified Glasgow Prognostic Score (MGPA); (Bottom) Comparative bar chart of misclassification rates between scoring systems.

Among cases with GCS scores ranging from 3 to 5, a notably higher percentage of patients (48.28%) did not survive compared to those who survived (2.96%), with a highly significant P-value of less than 0.0001. Similarly, for GCS scores of 6 to 12, the deceased group constituted 8.62% of

patients, while the survival rate was 2.8%. In contrast, the majority of cases with GCS scores between 13 and 15 survived (93.93%), demonstrating a substantial difference compared to the deceased percentage of 43.1%. There were similar obvious relationships between the triage level (P<0.0001), ISS (P<0.0001), and MGAP scale (P<0.0001) with mortality, as shown in Table-1. Cases mislabeled as medium or low risk that experienced death happened in 7 cases (of 598 available records; 1.17%) based on the ISS risk stratification system with 98.18% Positive Triage Accuracy and 44 cases (of 698 available records; 6.30%), based on the MGAP risk stratification system with 32.75% positive triage accuracy, as shown in Figure-1.

## Discussion

In the present study, the calculation of ISS and MGAP indicators has been done and these two indicators were compared with each other. In this study, a notable correlation was discerned between the triage level of the patients and the two indicators, in this way that the patients with triage level one (acute level) at the time of admission were in a higher category of ISS score and a lower category of MGAP score compared to level two and there were three triages.

Granström et al. in a study titled criteria-based protocol for the triage of trauma patients in the hospital, found that the correct prioritization of patients in the triage according to the score of ISS>15 for critically ill patients who need to receive more services and ISS<15 for patients with fewer symptoms that require fewer services, it can provide the possibility of improving patient care and allocating resources to meet his needs [12]. Lampi et al. in another study entitled the potential benefits of triage for trauma patients in the emergency department of Moi Hospital in Kenya and Cassignol et al. They concluded that the patients with the highest ISS category are in triage level (red level) [13][14]. As a result, when entering the triage, the higher the score of the ISS index and the lower the score of the MGAP index, the lower the triage level or, in other words, the more acute.

But, in case of assessment of the comparative accuracy of these triage tools, our study found that utilizing MGAP risk stratification system might cause a higher percentage of mislabeling patients that need acute care to low risk labels. Cases mislabeled as medium or low risk that experienced death happened in 7 cases (of 598 available records; 1.17%) based on the ISS risk stratification system with 98.18% Positive Triage Accuracy and 44 cases (of 698 available records; 6.30%), based on the MGAP risk stratification system with 32.75% positive triage accuracy.

While in study of Farzan et al. MGAP showed better mortality prediction accuracy in multiple trauma patients than the ISS [15]. But their study sample size was so smaller than ours. The size of the study population can influence the statistical power and reliability of the findings. Smaller sample sizes may result in greater variability in the observed outcomes. In cohort of 16,265 individuals by Larkin et al. [16] In this investigation, the TRISS exhibited the paramount predictive accuracy for mortality, with an area under the curve (AUC) of 0.920 (95% confidence interval: 0.911 to 0.929, P<0.0001). Subsequently, the MGAP scoring system closely trailed, manifesting an AUC of 0.900 (95% confidence interval: 0.889 to 0.911, P<0.0001). Conversely, the Injury Severity Score (ISS) and the New Injury Severity Score (NISS) displayed comparatively lower AUC values of 0.830 (95% confidence interval: 0.814 to 0.847) and 0.827 (95% confidence interval: 0.809 to 0.844), respectively. But in our study, we did not used these tools by continuous data and final categories were applied for estimations. As well as our study, when considering categorical view of the triage systems, Larkin et al. [16] study results were in agreement with our study. None of the scoring systems exhibited satisfactory sensitivity for forecasting mortality at historical thresholds, with sensitivity values spanning from 73% for ISS to 80% for NISS.

In the present study, there was no significant difference between the mean age of people placed in different categories of ISS, while there was a significant difference between the mean age of people placed in different categories of MGAP. Finally, we came to the conclusion that the most injured people were in the age group (18-44 years old) and it somehow indicated that young people are more at risk of injuries caused by trauma, which is similar to the article by Hamidreza Rihani et al. It is especially for multiple trauma patients [17]. This is while Yueh-Tzu Chiang et al., in the study of factors predicting the mortality of trauma patients examined by the trauma registry system, found that trauma patients aged 45 years and older were at a higher risk of mortality [18].

Mohammadreza Ahsaei and his colleagues in the study of using the ISS score to predict the survival of trauma patients in eastern Iran found the greatest risk of death for the age group of 21-40 years [19]. Hossein Abdali and Mehrdad Memarzadeh did not find a significant relationship between age and ISS score in their study under the title of injury severity assessment in trauma patients of Al-Zahra Medical Center [20]. Matthew S. Wilson et al., in a study titled Early Predictors of Mortality in Elderly Trauma Patients, found that older age was directly related to increased mortality [21]. Mohamed Amin Selim et al., in a study entitled MGAP score accuracy in forecasting mortality among multiple trauma patients, found that the percentage of patients who died at the age of 60 years and older was higher than at younger ages [22].

Our findings were compared with those of a study by Hajipoor Kashgsaray et al. (2024), which investigated the correlation between various severity scoring indices, including the Shock Index, GAP, RGAP, NTS, MGAP, MEWS, and TRISS, in predicting outcomes in moderate and severe trauma patients. Similar to our findings, the MGAP score was a significant predictor of morbidity and mortality. The study by Hajipoor Kashgsaray et al. found that an MGAP score less than 18 was the best cutoff point for predicting hospitalization in multi-trauma patients [23].

Our findings align with those of Mohammed et al. [24], who reported that the MGAP score had a high sensitivity (94%) in capturing the mortality subgroup, indicating its effectiveness in identifying critically ill patients. In contrast, Mohammed et al. found that the MGAP score had a good discriminatory ability with an AUROC of 0.890, which is slightly higher than the ISS's AUROC in our study. The higher AUROC for the MGAP score in their study suggests that it may be more effective in distinguishing between patients who will survive and those who will not, despite the lower positive triage accuracy observed in our study.

The differences in the performance of the scoring systems between our study and the study by Mohammed et al. can be attributed to the different healthcare settings and resource availability. Our study was conducted in a specialized trauma center in Iran, which likely has more advanced medical resources and a more structured triage system compared to the low-resource setting in Upper Egypt.

Our results are consonant with the investigation by Pourshaikhian et al. [25], which examined 8,000 multiply injured patients and documented a 3% fatality rate, with an average hospitalization duration of 3.7 ± 2 days. The prognostic efficacy of the ISS and the MGAP for predicting mortality and hospital stay duration was statistically robust (P<0.001), indicating a high level of confidence in their predictive power. The AUC values of 97.9% and 98.3% for ISS and MGAP, respectively, further underscore their exceptional accuracy in differentiating between patients with different outcomes. The association between MGAP and ISS for hospital stay duration was significant (r=-0.267 and r=-0.274, P<0.001), suggesting a moderate inverse relationship between the severity scores and the length of hospital stay.

However, the magnitude of this correlation between MGAP and ISS was not statistically meaningful, indicating that while there is a relationship, it is not strong enough to be considered a primary factor. The optimal threshold values for ISS and MGAP were determined to be 7 and 22.5, respectively. These cut-off points are crucial for clinical decision-making, as they help identify patients at higher risk. The sensitivity of 98.1% and 92.3% for ISS and MGAP, respectively, indicates a high ability to correctly identify patients who will have the outcomes of interest. The specificity of 96.7% and 92.3% suggests a strong ability to correctly identify patients who will not have the outcomes of interest. The PPV of 97.7% and 92.3% further supports the reliability of these scores in predicting positive outcomes, while the NPV of 92.3% and 98.1% indicates a high confidence in ruling out negative outcomes.

In our study, the majority of patients (81%) were male, and the overall survival rate was 92%. These findings are consistent with the Yadollahi et al. study [26], which also reported a higher proportion of male patients and a survival rate of 90.3% (1681 out of 1861 patients).

Both studies found that age was a significant predictor of mortality, with older patients having a higher risk of death. However, our study found that gender, trauma reason, and race did not significantly affect mortality, which aligns with the findings of Yadollahi et al. In our study, the ISS and MGAP scoring systems were both found to be significant predictors of mortality, with P-values<0.0001. The ISS was particularly effective in stratifying patients into different risk categories, with a high positive triage accuracy of 98.18%. However, the MGAP system had a lower positive triage accuracy of 32.75%, indicating that it mislabeled a higher proportion of patients as low or medium risk who ultimately died. Yadollahi et al. reported that the AUC for the ISS was 0.88, which is slightly lower than the AUC of 0.9 for the MGAP system. This suggests that, in their study, the MGAP system had a marginally better overall accuracy in predicting mortality compared to the ISS. The differences in the performance of the ISS and MGAP scoring systems between our study and the study by Yadollahi et al. can be attributed to a combination of factors, including sample size, demographic characteristics, study setting, scoring system application, data quality, clinical practices, and methodological differences.

## Conclusion

The study's conclusive observations shows the predictive efficacy of ISS, revealing a commendably low mislabeling rate coupled with a high level of triage accuracy. This implies that ISS serves as a reliable and precise tool for assessing the severity of trauma in multiple trauma patients, fostering confidence in its applicability across diverse clinical scenarios. On the contrary, the MGAP risk stratification system shows a comparatively higher mislabeling rate, indicating instances where its application might lead to inaccurate results. This discrepancy suggests that the efficacy of MGAP is nuanced and may be influenced by specific contextual factors, rendering its universal applicability across all scenarios questionable. Further research and validation studies are warranted to elucidate the contexts where MGAP may excel and where alternative scoring systems may be more adept, ultimately contributing to informed decision-making in the complex realm of multiple trauma management.

## Conflict of Interest

The authors declare no conflicts of interest relevant to this study.
